# Eficácia de Metodologia Ativa de Aprendizagem do ECG no Internato em Clínica Médica

**DOI:** 10.36660/abc.20220446

**Published:** 2022-11-09

**Authors:** Márcia Cristina Amélia da Silva, Maria Elisa Lucena Sales de Melo Assunção

**Affiliations:** 1 Uninassau Unidade Boa Viagem Recife PE Brasil Centro Universitário Maurício de Nassau ( Uninassau ) - Curso de Medicina, Unidade Boa Viagem , Recife , PE – Brasil; 2 Centro Médico-Hospitalar da Polícia Militar de Pernambuco Coordenação do Internato Médico Uninassau Recife PE Brasil Centro Médico-Hospitalar da Polícia Militar de Pernambuco - Coordenação do Internato Médico , Uninassau - Departamento de Clínica Médica, Recife , PE – Brasil; 3 Hospital Geral de Areias Secretaria Estadual de Saúde-PE Recife PE Brasil Hospital Geral de Areias / Secretaria Estadual de Saúde-PE , Recife , PE – Brasil

**Keywords:** Eletrocardiografia, Aprendizagem Baseada em Problemas, Educação Médica

## Abstract

**Fundamento:**

Estudos têm mostrado uma baixa acurácia de médicos urgentistas em interpretar o eletrocardiograma (ECG) em quadros cardiológicos graves.

**Objetivo:**

Avaliar a eficácia de um método de aprendizagem do ECG no internato de clínica médica e conhecer a percepção dos internos quanto ao aprendizado antes e após a metodologia.

**Métodos:**

Foi utilizado um banco de dados com os resultados do pré e pós-teste de ECG das turmas de internato em clínica médica de 2017 a 2022. Foi enviado um questionário qualitativo com perguntas para autoavaliação da percepção do aprendizado.

**Resultados:**

Foram incluídos 227 estudantes, 161 (70,9%) do sexo feminino, com idade de 26,4 ± 4,2 anos. A média do pré-teste foi 3,75 ± 2,0 pontos e do pós-teste 8,48 ± 1,5 pontos, diferença estatisticamente significativa, mesmo após estratificação por sexo, idade e período do curso (p < 0,001 para todas as comparações). Sessenta e nove (30%) dos alunos responderam ao questionário qualitativo. Os três sentimentos predominantes anteriores ao aprendizado foram desespero, medo e insegurança. Após o Clube, os sentimentos predominantes foram segurança, tranquilidade e confiança.

**Conclusão:**

Foi baixo o nível de conhecimento prévio do ECG entre os egressos do internato médico e a metodologia proposta foi eficaz em melhorar o grau de conhecimento do ECG, independente da idade, sexo ou período do curso. E foi possível transformar as crenças negativas quanto à aprendizagem do ECG e torná-la significativa e leve. Um olhar mais incisivo nos cursos de medicina para o aprendizado do ECG de forma mais prática e contextualizada, pode melhorar este cenário.

## Introdução

As urgências e emergências cardiológicas correspondem a até 10% dos atendimentos ^1^ e é o segundo motivo mais comum pelo qual um adulto procura a emergência clínica nos Estados Unidos. ^2^ Portanto, a importância do conhecimento da eletrocardiografia na urgência clínica é inquestionável. Estudos têm mostrado uma baixa acurácia de médicos urgentistas em interpretar o eletrocardiograma (ECG) de doenças graves como infarto, arritmias ventriculares e bloqueios atrioventriculares avançados. ^3 - 5^ E muitos egressos do curso médico referem-se como inseguros no atendimento a pacientes cardiológicos com o conteúdo aprendido na graduação. Entretanto, faltam estudos que avaliem a eficácia de metodologias no ensino do ECG nesta etapa da formação médica, o ciclo prático do internato, que antecede a sua formação como médicos. Este estudo tem o objetivo de avaliar a eficácia de um método de ensino do ECG no internato de clínica médica a estudantes do 9º ao 12º período, de um centro universitário privado, usando uma técnica inovadora, de metodologia ativa, já descrita anteriormente e conhecer a autoavaliação dos estudantes quanto ao seu conhecimento antes e após aplicação da técnica. ^6^

## Métodos

### Tipo de estudo

Estudo observacional quantitativo, do tipo antes e depois, dos resultados das avaliações dos internos em clínica médica e análise qualitativa da percepção dos internos sobre o antes e depois.

### População do estudo

Estudantes do 9º ao 12º período de medicina do centro universitário privado da cidade de Recife, PE, Brasil. Seleção e tamanho amostral por conveniência.

### Período do Estudo

Março 2017 a maio 2022.

### Metodologia do Clube do ECG

O rodízio de clínica médica da instituição tem a duração de 12 semanas e é realizado em 3 centros de médio porte. Como parte do conteúdo teórico do rodízio está o Clube do ECG, que permeia as 12 semanas, com encontros semanais de 2 horas.O preceptor responsável pela condução do Clube de ECG é o coordenador local do rodízio nas duas Instituições e ambos são cardiologistas de formação, seguindo a mesma metodologia de ensino do ECG.

A metodologia utilizada para o ensino do ECG foi descrita em publicação da Revista da Associação Brasileira de Ensino Médico ^6^ e consiste em 8 encontros assim distribuídos: 2 aulas expositivas dialogadas que apresentam o mnemônico REFASA ou RIFEMOS, como atualmente o chamamos. Inicialmente, o mnemônico era o REFASA, isto é, Ritmo, Eixo, Frequência, Alterações morfológicas de P, Pri e QRS, S do ST e T. O último A do mnemônico era para “Alterações outras” e incluía a análise de alterações patológicas outras, como onda Q. Atualmente usamos o RIFEMOS, pois invertemos a ordem de análise do eixo para depois da frequência cardíaca após percepção dos internos de que era mais importante ver a frequência cardíaca para definição das taquiarritmias supraventriculares. No RIFEMOS, o RI = ritmo, F = frequência cardíaca, E = eixo cardíaco, MO = análise morfológica das ondas P, Pri e QRS, S = segmento ST e onda T. Após compreenderem o método, os internos foram divididos em 6 pequenos grupos e a cada semana, um grupo foi responsável por apresentar a interpretação de um número de traçados enviado na semana anterior para todo o grupo, usando o mnemônico. A preceptoria deu o contexto clínico após a apresentação de cada traçado e corrigiu possíveis entendimentos equivocados. Todos deveriam estar com os traçados impressos em tamanho A4 e foram questionados a participarem ativamente das dúvidas. Cada clube de ECG foi identificado como Clube 1, 2, 3, 4, 5 e 6 e correspondia a um tema específico, assim especificados: 1 = distúrbios de condução intraventricular; 2 = sobrecargas de câmaras; 3 = distúrbios de condução atrioventricular; 4 = alterações do ST-T; 5 = taquiarritmias supraventriculares regulares; 6 = taquiarritmias supraventriculares irregulares e taquicardia ventricular.

Foi utilizado o banco de dados com as notas dos pré e pós-teste dos internos de medicina de clínica médica de duas das três instituições onde os internos realizam o seu treinamento de clínica médica. O pré-teste foi constituído de questões de casos clínicos de cardiologia de urgência e emergência, cujo diagnóstico do ECG é a base para a conduta clínica. Foi aplicado na segunda semana do rodízio e reaplicado como pós-teste na última semana. A nota obtida no pós-teste foi utilizada como parte da nota teórica do rodízio.

### Metodologia da pesquisa

Na etapa retrospectiva, foi realizada busca no banco de dados dos resultados do pré e pós-teste das turmas de 2017 a 2022, além das informações como idade, sexo e período em que cursou clínica médica.

Na etapa prospectiva, foi enviado questionário qualitativo para todos os participantes com as seguintes perguntas:

1. Você considera que o aprendizado do Clube de ECG foi: útil, necessário, desnecessário para o rodízio e sua prática, inútil para sua prática, atrapalhou seu aprendizado de clínica médica, contribuiu com seu aprendizado de clínica médica, desmistificou o seu aprendizado de ECG, contribuiu com suas crenças de que ECG é difícil, fez o ECG ser mais fácil e simples.
2. Descreva com uma frase o que você sentia antes do Clube ao receber um ECG nas mãos.
3. Descreva com uma frase o que você sente hoje ao receber um ECG nas mãos.

### Análise estatística dos dados quantitativos

Os dados categóricos foram resumidos através de frequências absolutas e relativas. Os dados numéricos foram resumidos através da média e desvio-padrão, por terem os dados distribuição normal (teste de Shapiro). Comparação entre as médias do pré e pós-testes foi feita usando o teste T de Student para amostras pareadas. Em todos os testes foi adotado o nível de significância (valor de p) de 0,05 com intervalo de confiança (IC) de 95%. Os dados foram analisados pelo programa Stata 12.0 (Stata, College Station, Texas, EUA).

### Análise qualitativa

A análise qualitativa foi através da técnica de análise de conteúdo do discurso sobre a percepção do aprendizado do ECG.

## Resultados

Na análise quantitativa foram incluídos todos os alunos que rodaram a clínica médica no período do estudo, um total de 227 estudantes, dos quais 161 (70,9%) era do sexo feminino e a média de idade de 26,4 ± 4,2 anos. A Tabela 1 apresenta as características gerais dos pesquisados.

**Tabela 1:** Característica dos pesquisados

Características	Número (%)
**Idade (mínimo – máximo)**	22 – 44 anos
**Faixa etária**	
Até 24 anos	76 (33,5%)
De 25 a 29 anos	110 (48,5%)
30 anos e mais	41 (18,0%)
**Período**	
Nono	51 (23,8%)
Décimo	60 (26,4%)
Décimo primeiro	70 (30,9%)
Décimo segundo	43 (18,9%)

As notas do pré-teste foram em 84,5% (192) abaixo de 6,0 pontos, com média geral de 3,75 ± 2,0 pontos. As médias do pré-teste não apresentaram diferenças estatisticamente significativas quanto ao sexo, idade ou período que cursava o interno ao participar do Clube de ECG.

As notas dos pós-testes foram 68,5% (155) acima de 8,0 pontos e 84% (190) acima de 7,0 pontos. O incremento médio à nota do pré-teste foi de 4,73 pontos (IC 95% 4,46 – 4,99), não havendo também diferenças estatisticamente significativas nos resultados quanto ao sexo, idade ou período que cursava o interno ao participar do Clube de ECG.

A diferença entre as notas do pré e pós-testes foi estatisticamente significativa, mesmo após estratificação por sexo, idade e período do curso (p < 0,001 para todas as comparações). E o ganho médio de nota no pós-teste foi semelhante para todos os participantes, não havendo diferença estatisticamente significativa quando comparados por sexo, idade ou período cursado. A Tabela 2 apresenta os resultados das comparações das notas do pré e pós-teste de todos os participantes e estratificação por idade, sexo e período.

**Tabela 2:** Comparação das notas pré e pós teste ECG

Características	Pré-teste (média ± DP)	Pós-teste (média ± DP)	Ganho médio de nota no pós-teste (IC 95%)	p-valor
**Geral**	3,75 ± 2,0	8,48 ± 1,5	4,73 (4,46 – 4,99)	<0,001
**Sexo**				
Feminino	3,76 ± 2,0	8,53 ± 1,5	4,77 (4,45 – 5,09)	<0,001
Masculino	3,73 ± 1,8	8,37 ± 1,3	4,63 (4,16 – 5,10)	<0,001
p-valor	0,865	0,479	0,640	-
**Faixa etária**				
Até 24 anos	3,68 ± 1,8	8,55 ± 1,5	4,87 (4,48 – 5,26)	<0,001
De 25 a 29 anos	3,80 ± 2,1	8,58 ± 1,4	4,79 (4,36 – 5,21)	<0,001
30 anos e mais	3,75 ± 2,1	8,07 ± 1,6	4,32 (3,72 – 4,92)	<0,001
p-valor	0,924	0,134	0,346	-
**Período**				
Nono	3,53 ± 1,9	8,45 ± 1,8	4,91 (4,29 – 5,54)	<0,001
Décimo	3,73 ± 1,9	8,33 ± 1,4	4,59 (4,13 – 5,07)	<0,001
Décimo primeiro	3,77 ± 2,0	8,56 ± 1,4	4,79 (4,30 – 5,27)	<0,001
Décimo segundo	4,02 ± 2,1	8,60 ± 1,9	4,58 (3,98 – 5,19)	<0,001
**p-valor**	0,686	0,767	0,807	-

DP: desvio padrão; IC: intervalo de confiança.

Dos 227 participantes, 69 (30,5%) responderam ao questionário da pesquisa qualitativa. A média de idade dos respondedores foi de 25,6 ± 3,5 anos e 49 (71%) era do sexo feminino. Não houve diferença estatisticamente significativa entre as características idade e sexo entre respondedores e a amostra total do estudo, bem como quanto às médias do pré e pós-teste (3,28 ± 1,8 e 8,45 ± 2,2, respectivamente).

Na pergunta fechada “ *Você considera que o aprendizado do Clube de ECG foi* ” 60 (86,9%) responderam que foi útil, 55 (79,7%) que contribuiu com o aprendizado de clínica médica, 61 (88,4%) que tornou o aprendizado do ECG mais fácil e simples e 64 (92,7%) que o método desmistificou o aprendizado do ECG.

Na pergunta aberta *“Descreva com uma frase o que você sentia antes do clube ao receber um ECG nas mãos”* , os três sentimentos predominantes foram desespero, medo e insegurança. Já na segunda pergunta aberta *“Descreva com uma frase o que você sente hoje ao receber um ECG nas mãos”* , os sentimentos predominantes foram segurança, tranquilidade e confiança. Na Tabela 3 , as categorias extraídas dos discursos dos internos nas questões abertas.

**Tabela 3:** Categorias após análise do discurso dos alunos sobre sua percepção quanto à aprendizagem do ECG antes e depois do Clube

Antes do Clube	Após o Clube
Desespero	Confiança
Eu sentia pânico de não saber interpretar	Mais tranquilidade
Medo de errar	Ainda preciso aprender muito, mas me sinto confiante
Aflição	Descobri que sei ver um ECG
Nervosismo e falta de segurança	Maior segurança para interpretação e raciocínio clínico do paciente
Desespero e tristeza	Entusiasmo
Medo e preocupação	Eu perdi o medo do ECG
Não entendia ECG	Mais seguro para diagnosticar as principais alterações no ECG em ambiente de UTI e emergência
Terror	Feliz em poder discutir sobre ECG
ECG é difícil	ECG é fácil
Nunca vou aprender	Segurança para ler o ECG e identificar alterações
Incompetência	Consigo interpretar com clareza o ECG
Insegurança	Mais seguro
Me sentia incapaz e perdida	Eu fico animada para interpretar, o desafio passou a ser muito bom!
Desespero e angústia	Me sinto menos angustiado e que hoje estou conseguindo entender o que não está certo no ECG
Me sentia incapaz e perdida	Mais segurança no diagnóstico e tratamento
Ficava nervoso, pois não conseguia interpretá-lo	Me sinto capaz de analisar e identificar alterações importantes
Não me sentia preparado para ser médico	Alívio de poder laudar um ECG e assim salvar vidas!

ECG: eletrocardiograma; UTI: unidade de terapia intensiva.

## Discussão

A pesquisa mostrou um baixo conhecimento de ECG pelos estudantes, independentemente de ser o seu primeiro contato com o internato (estudantes do 9º período) ou já estarem próximos à formatura (12º período), com média abaixo de 5,0 para ambos os grupos (3,53 versus 4,02 pontos). Este achado corrobora os achados de pesquisa realizada na UNIFESO em 2003 ^7^ com estudantes do 8º período de medicina desta universidade, período que antecede a entrada no ciclo do internato médico. Nenhum estudo brasileiro ou internacional avaliou estudantes no ciclo do internato quanto ao seu grau de conhecimento em ECG.

O resultado de aprendizagem demonstrado pela metodologia RIFEMOS na média do pós-teste (8,48 ± 1,5 pontos), com incremento médio significativo e independente da idade ou período do aluno, de 4,73 pontos (IC 95% 4,46 – 4,99) demonstra quão importante é considerar a inserção deste modelo de aprendizagem no internato, quando o aluno tem uma perspectiva clínica-assistencial mais desenvolvida do que durante a graduação. Nenhum estudo foi encontrado na literatura nacional ou internacional que tenha realizado comparação semelhante, em internos de medicina.

Considerar os sentimentos dos alunos quanto ao exame e torná-los conscientes destes sentimentos, foi o primeiro passo para que a realidade deles fosse modificada por eles mesmos. Assim, foi possível fazê-los acreditar e gostar de estudar ECG, tornando-se independentes para a aprendizagem permanente e por toda a vida. A modificação de percepção dos alunos após o Clube de ECG foi evidente em todas as falas. A combinação de aprendizagem baseada em casos reais de ECG com problemas clínicos comuns nas emergências trouxe para a realidade do rodízio de clínica médica e de sua prática futura como urgentistas a motivação extrínseca para o aprendizado. O resultado do pré-teste ativou a motivação interna de cada um. Este resultado é semelhante ao encontrado por Zhao et al. no ensino da interpretação e abordagem de doenças da tireoide, com estudantes do 4º ano e residentes de clínica na China. ^8^ Segundo os autores, este modelo de aprendizagem aumenta a motivação para aprender, compreensão, interação aluno-professor, resultados no exame final, habilidades de comunicação, habilidades de pensamento clínico, habilidades de autoaprendizagem, habilidades de trabalho em equipe e absorção de conhecimento. Habilidades estas extremamente necessárias para a prática médica, notadamente na emergência clínica.

O paradigma quanto à aprendizagem do ECG vem de longa data e permeia a formação médica de muitos dos atuais docentes e cardiologistas das escolas médicas. Entretanto, se faz necessária a desmistificação da aprendizagem deste método tão valioso e tão simples, de baixo custo e amplamente disponível nas emergências. Portanto, é urgente a revisão das escolas médicas quanto à metodologia aplicada no ensino na graduação ou mesmo no internato.

As limitações do estudo são: a natureza observacional e retrospectiva do estudo quantitativo, a menor amostra para a análise qualitativa e o fato de ter sido o estudo realizado em um único centro universitário.

Mais estudos na área do ensino do ECG devem ser incentivados pelas sociedades acadêmicas e médicas, visando ampliar o conhecimento dos egressos dos cursos médicos neste exame tão essencial nas urgências e emergências médicas.

## Conclusão

Esta pesquisa demonstrou baixo nível de aprendizado do ECG entre os egressos do internato médico e a eficácia da metodologia proposta quanto ao aprendizado da interpretação do ECG, independente da idade, sexo ou período do curso. E que é possível, em pouco tempo, com metodologia simples, modificar as crenças dos alunos quanto ao ECG e converter isto em aprendizagem sólida, contextualizada e útil para a prática médica.
