# Supplementary material for: Eficácia de Metodologia Ativa de Aprendizagem do ECG no Internato em Clínica Médica
Source: Arq Bras Cardiol. 2022 Nov 9;119(5 Suppl 1):22–6. [Article in Portuguese] doi: 10.36660/abc.20220446 (PMC9750196; doi:10.36660/abc.20220446)
Supplement: Translation

# Translation: Effectiveness of an Active Methodology for Learning ECG during the Internal Medicine Internship

**Authors:** Márcia Cristina Amélia da Silva, Maria Elisa Lucena Sales de Melo Assunção

**Affiliations:** 1 Brazil Centro Universitário Maurício de Nassau (Uninassau) - Curso de Medicina, Unidade Boa Viagem, Recife, PE – Brazil; 2 Brazil Centro Médico-Hospitalar da Polícia Militar de Pernambuco - Coordenação do Internato Médico, Uninassau - Departamento de Clínica Médica, Recife, PE – Brazil; 3 Brazil Hospital Geral de Areias/Secretaria Estadual de Saúde-PE, Recife, PE – Brazil

**Keywords:** Electrocardiography, Problem-Based Learning, Education, Medical

## Abstract

**Background:**

Studies have shown a low accuracy of urgent care physicians in interpreting electrocardiogram (ECG) in severe cardiovascular conditions.

**Objective:**

To evaluate the effectiveness of an ECG learning method in internal medicine internships and to know the perception of interns regarding learning before and after the methodology.

**Methods:**

This study used a database with the results of an ECG pre- and post-test of the classes in the internal medicine internship from 2017 to 2022. A qualitative questionnaire was sent with questions for self-assessment of perception of learning.

**Results:**

The study included a total of 227 students, 161 of whom (70.9%) were female. The mean age was 26.4 ± 4.2 years old. The pre-test mean was 3.75 ± 2.0 points, and the post-test mean was 8.48 ± 1.5 points, showing a statistically significant difference, even after stratification by sex, age, and course period (p < 0.001 for all comparisons). Sixty-nine (30%) of the students responded to the qualitative questionnaire. The three predominant feelings prior to learning were despair, fear, and insecurity. After the Club, the predominant feelings were security, tranquility, and confidence.

**Conclusion:**

The level of prior knowledge regarding ECG was low among students in the medical internship, and the proposed methodology was effective for learning ECG, regardless of age, sex, or course period. It was possible to transform negative beliefs regarding ECG learning and make learning meaningful and enjoyable. A more incisive look at medical courses for learning the ECG in a more practical and contextualized way can improve this scenario.

## Introduction

Cardiovascular urgencies and emergencies correspond to up to 10% of consultations,^1^ and they are the second most common reason for adults seeking emergency clinics in the United States.^2^ Therefore, the importance of knowledge of electrocardiography in clinical urgencies is unquestionable. Studies have shown a low accuracy of emergency physicians in interpreting electrocardiograms (ECG) of serious diseases, such as infarction, ventricular arrhythmias, and advanced atrioventricular blocks.^3 - 5^ Many students who graduate from medical school report that they are insecure in caring for cardiac patients with the content learned during their course of studies. However, there is a lack of studies that evaluate the effectiveness of methodologies for teaching ECG at this stage of medical training, the practical cycle of internship, which precedes their training as doctors. The objectives of this study were to evaluate the effectiveness of an ECG teaching method in the internal medicine internship for students from the ninth to the twelfth period, in a private university center, using an innovative technique, with active methodology, which has previously been described, and to know the self-assessment of students regarding their knowledge before and after the application of the technique.^6^

## Methods

### Study type

This was a before-and-after quantitative observational study of the results of interns’ evaluations in internal medicine, with qualitative analysis of the interns’ perceptions before and after.

### Study population

The study included students from the ninth to the twelfth period of medicine at a private university center in the city of Recife, Pernambuco, Brazil, with sample size and selection by convenience.

### Study period

March 2017 to May 2022.

### Methodology of the ECG Club

The institution’s internal medicine rotation lasts for 12 weeks, and it takes place in 3 medium-sized centers. The ECG Club is part of the theoretical content of the rotation, and it occurs throughout the 12 weeks, with weekly 2-hour meetings. The preceptor responsible for guiding the ECG Club is the local coordinator of the rotation, cardiologists, and the same methodology is followed.

The methodology used for teaching ECG has been described in a publication in the *Revista da Associação Brasileira de Ensino Médico* ,^6^ and it consists of 8 meetings distributed in the following manner: 2 lecture classes with dialogue that introduce the Portuguese mnemonic devices, REFASA or RIFEMOS, as we currently call it. Initially, the mnemonic was REFASA, which stood for rhythm, axis, heart rate, morphological changes of P, Pri and QRS, S, ST, and T. The last A of the mnemonic stood for “other changes” including analysis of other pathological changes, such as the Q wave. We currently use RIFEMOS, as we reversed the order of analysis with axis after heart rate, following the interns’ perception that it was more important to see the heart rate for definition of supraventricular tachyarrhythmias. In RIFEMOS, RI = rhythm, F = heart rate, E = cardiac axis, MO = morphological analysis of P, Pri, and QRS waves, S = ST segment and T wave. After understanding the method, the interns were divided into 6 small groups, and each week, 1 group was responsible for presenting the interpretation of a number of tracings sent during the previous week to the entire group, using the mnemonic. The preceptor provided the clinical context after the presentation of each tracing and corrected possible misunderstandings. All students were expected to have their tracings printed in A4 size, and they were encouraged to actively participate regarding their doubts. Each ECG Club meeting was identified as Club 1, 2, 3, 4, 5, and 6, corresponding to a specific theme, as follows: 1 = intraventricular conduction disorders; 2 = chamber overloads; 3 = atrioventricular conduction disorders; 4 = ST-T changes; 5 = regular supraventricular tachyarrhythmias; 6 = irregular supraventricular tachyarrhythmias and ventricular tachycardia.

The study used the database with pre- and post-test scores of internal medicine interns from 2 of the 3 institutions where interns perform their internal medicine training. The pre-test consisted of questions from clinical cases of urgent and emergency cardiology, where ECG diagnosis is the basis for clinical management. It was applied in the second week of the rotation and reapplied as a post-test in the final week. The score obtained on the post-test was included as part of the theoretical grade for the rotation.

### Research methodology

During the retrospective stage, a search was carried out in the database of the results of the pre- and post-tests of the classes from 2017 to 2022, in addition to information such as age, sex, and period when they were enrolled in internal medicine.

During the prospective stage, a qualitative questionnaire was sent to all participants, with the following questions:

Do you consider that the learning experience in the ECG Club: was useful, was necessary, was unnecessary for rotation and your practice, was useless for your practice, interfered with your learning of internal medicine, contributed to your learning of clinical medicine, demystified your learning of ECG, contributed to your belief that ECG is difficult, made ECG easier and simpler?

In a single sentence, describe how you felt before the Club when receiving an ECG in your hands.

In a single sentence, describe how you feel today when receiving an ECG in your hands.

### Statistical analysis of quantitative data

Categorical data were summarized using absolute and relative frequencies. Numerical data were summarized using mean and standard deviation, as the data had a normal distribution (Shapiro test). Comparison between pre- and post-test means was performed using Student’s T test for paired samples. In all tests, a significance level (p value) of 0.05 and 95% confidence interval (CI) were adopted. Data were analyzed using the Stata 12.0 program (Stata, College Station, Texas, USA).

### Qualitative analysis

Qualitative analysis was performed using the technique of speech content analysis regarding the perception of ECG learning.

## Results

The quantitative analysis included all students who completed the internal medicine rotation during the study period, a total of 227 students, 161 of whom (70.9%) were female. The mean age was 26.4 ± 4.2 years old. Table 1 exhibits the general characteristics of the research participants.

**Table 1:** Characteristics of the study participants

Characteristics	Number (%)
**Age (minimum to maximum)**	22 to 44 years
**Age range**	
24 years and younger	76 (33.5%)
From 25 to 29 years	110 (48.5%)
30 years and older	41 (18.0%)
**Period**	
Ninth	51 (23.8%)
Tenth	60 (26.4%)
Eleventh	70 (30.9%)
Twelfth	43 (18.9%)

Regarding pre-test scores, 84.5% (192) were below 6.0 points, with an overall average of 3.75 ± 2.0 points. The pre-test averages did not show statistically significant differences regarding sex, age, or period enrolled in the internship when participating in the ECG Club.

Regarding post-test scores, 68.5% (155) were above 8.0 points, and 84% (190) were above 7.0 points. The average increment from the pre-test score was 4.73 points (95% CI 4.46 – 4.99), and there were no statistically significant differences in the results regarding sex, age, or period enrolled in the internship when participating in the ECG Club.

The difference between pre- and post-test scores was statistically significant, even after stratification by sex, age, and course period (p < 0.001 for all comparisons). The average post-test score gain was similar for all participants, with no statistically significant differences when comparing by sex, age, or course period. Table 2 exhibits the results of the comparisons of pre- and post-test scores for all participants and stratification by age, sex, and course period.

**Table 2:** Comparison of ECG pre- and post-test scores

Characteristics	Pre-test (mean ± SD)	Post-test (mean ± SD)	Mean score gain on the post-test (95% CI)	p value
**General**	3.75 ± 2.0	8.48 ± 1.5	4.73 (4.46 – 4.99)	<0.001
**Sex**				
Female	3.76 ± 2.0	8.53 ± 1.5	4.77 (4.45 – 5.09)	<0.001
Male	3.73 ± 1.8	8.37 ± 1.3	4.63 (4.16 – 5.10)	<0.001
p value	0.865	0.479	0.640	-
**Age range**				
24 years and younger	3.68 ± 1.8	8.55 ± 1.5	4.87 (4.48 – 5.26)	<0.001
From 25 to 29 years	3.80 ± 2.1	8.58 ± 1.4	4.79 (4.36 – 5.21)	<0.001
30 years and older	3.75 ± 2.1	8.07 ± 1.6	4.32 (3.72 – 4.92)	<0.001
p value	0.924	0.134	0.346	-
**Period**				
Ninth	3.53 ± 1.9	8.45 ± 1.8	4.91 (4.29 – 5.54)	<0.001
Tenth	3.73 ± 1.9	8.33 ± 1.4	4.59 (4.13 – 5.07)	<0.001
Eleventh	3.77 ± 2.0	8.56 ± 1.4	4.79 (4.30 – 5.27)	<0.001
Twelfth	4.02 ± 2.1	8.60 ± 1.9	4.58 (3.98 – 5.19)	<0.001
**p value**	0.686	0.767	0.807	-

*CI: confidence interval; SD: standard deviation.*

Of the 227 participants, 69 (30.5%) responded to the qualitative research questionnaire. The mean age of respondents was 25.6 ± 3.5 years, and 49 (71%) were female. There were no statistically significant differences for the characteristics of age and sex between respondents and the total study sample, or between the pre- and post-test means (3.28 ± 1.8 and 8.45 ± 2.2, respectively).

To the closed question (“ *Do you consider that the learning experience in the ECG Club* ”), 60 (86.9%) answered that it was useful, 55 (79.7%) that it contributed to the learning of internal medicine, 61 (88.4%) that it made learning ECG easier and simpler, and 64 (92.7%) that the method demystified ECG learning.

For the open-ended question, “ *In a single sentence, describe how you felt before the Club when receiving an ECG in your hands* ”, the 3 predominant feelings were despair, fear, and insecurity. For the second open-ended question, “ *In a single sentence, describe how you feel today when receiving an ECG in your hands* ”, the predominant feelings were security, tranquility, and confidence. Table 3 exhibits the categories extracted from the speeches of the interns in the open-ended questions.

**Table 3:** Categories after analysis of students’ speech about their perception of learning ECG before and after the Club

Before the Club	After the Club
Despair	Confidence
I felt panic about not knowing how to interpret it	More tranquility
Fear of making a mistake	I still have a lot to learn, but I feel confident
Affliction	I discovered that I know how to look at an ECG
Nervousness and insecurity	More security for interpretation and clinical reasoning for patients
Despair and sadness	Enthusiasm
Fear and worry	I overcame the fear of ECG
I didn’t understand ECG	Greater security to diagnose the main ECG changes in the intensive care unit and emergency environment
Terror	Happy to be able to discuss ECG
ECG is difficult	ECG is easy
I’m never going to learn	Security to read ECG and identify changes
Incompetence	I can interpret ECG clearly
Insecurity	More security
I felt unable and lost	I am excited to interpret. The challenge has become very good!
Despair and anguish	I feel less distressed and today I am able to understand what is not right on the ECG
I felt unable and lost	More security in diagnosis and treatment
I felt nervous because I couldn’t interpret it	I feel able to analyze and identify important changes
I didn’t feel prepared to be a doctor	Relief to be able to read an ECG and save lives!

*ECG: electrocardiogram.*

## Discussion

This research has shown a low level of knowledge of ECG by students, regardless of whether it was their first contact with the internship (students in the ninth period) or they were already close to graduating (twelfth period), with an average score below 5.0 for both groups (3.53 versus 4.02 points). This finding corroborates the findings of a study carried out in 2003 at UNIFESO^7^ with students in the eighth period of medicine at the university, where this period precedes entry into the medical internship cycle. No Brazilian or international study has evaluated students in the internship cycle regarding their level of knowledge about ECG.

The learning result demonstrated by the RIFEMOS methodology in the post-test average (8.48 ± 1.5 points), with a significant average increase, regardless of students’ age or period, of 4.73 points (95% CI 4.46 to 4.99) demonstrates how important it is to consider the inclusion of this learning model in the internship, when students have a more developed clinical-care perspective than during undergraduate studies. No study was found in the Brazilian or international literature that performed a similar comparison in medical interns.

Considering the students’ feelings about the exam and making them aware of these feelings was the first step to making it possible for them to modify their own reality. Thus, it was possible to help them feel confident and enjoy studying ECG, promoting independence for permanent, lifelong learning. The change in students’ perception after the ECG Club was evident in all speeches. The combination of learning based on real ECG cases with clinical problems common in emergencies brought the extrinsic motivation for learning to the reality of the internal medicine rotation and of their future practice as urgent care workers. The result of the pre-test activated each student’s internal motivation. This result is similar to that found by Zhao et al. in teaching the interpretation and approach of thyroid diseases, with fourth year students and clinical residents in China.^8^ According to the authors, this learning model increases motivation to learn, understanding, student-professor interaction, final exam results, communication skills, clinical thinking skills, self-learning skills, teamwork skills, and knowledge absorption. These skills are extremely necessary for medical practice, especially in clinical emergencies.

The paradigm regarding ECG learning has existed for a long time, and it permeates the medical training of many of the current professors and cardiologists in medical schools. However, it is necessary to demystify the learning of this highly valuable, simple, and low-cost method, which is widely available in emergencies. Therefore, it is urgent to review medical schools regarding the methodology applied in teaching during undergraduate studies or even internships.

The study limitations include the observational and retrospective nature of the quantitative study, the smaller sample size for the qualitative analysis, and the fact that the study was carried out in a single university center.

More studies in the area of ECG teaching should be encouraged by academic and medical societies, with the aim of expanding the knowledge of students graduating from medical courses on this exam, which is highly essential in medical urgencies and emergencies.

## Conclusion

This study found a low level of ECG learning among students in the medical internship, and it demonstrated the effectiveness of the proposed methodology regarding learning to interpret ECG, regardless of age, sex, or course period. It is possible, in a short time, with a simple methodology, to modify students’ beliefs about ECG and convert them into learning that is solid, contextualized, and useful for medical practice.
